# Lactic Acid Bacteria Metabolites Modulate Immune Response Against *Staphylococcus haemolyticus*-Infected RAW264.7 Murine Macrophage: A Novel Approach for Bovine Mastitis

**DOI:** 10.3390/ani15223338

**Published:** 2025-11-19

**Authors:** Nitsanat Cheepchirasuk, Sureeporn Suriyaprom, Thida Kaewkod, Varachaya Intachaisri, Thararat Chitov, Aussara Panya, Witaya Suriyasathaporn, Yingmanee Tragoolpua

**Affiliations:** 1 Department of Biology, Faculty of Science, Chiang Mai University, Chiang Mai 50200, Thailand; nitsanat_cheep@cmu.ac.th (N.C.); thida.kaewkod@cmu.ac.th (T.K.); thararat.chitov@cmu.ac.th (T.C.); aussara.pan@cmu.ac.th (A.P.); 2 Office of Research Administration, Chiang Mai University, Chiang Mai 50200, Thailand; sureeporn.suriyaprom@cmu.ac.th (S.S.); varachaya.int@cmu.ac.th (V.I.); 3 Faculty of Veterinary Medicine, Chiang Mai University, Chiang Mai 50100, Thailand; suriyasathaporn.witaya.y3@f.mail.nagoya-u.ac.jp; 4 Overseas Division Cambodia Office, Asian Satellite Campus Institute, Nagoya University, Nagoya 464-0813, Japan

**Keywords:** bovine mastitis, immune response, *S. haemolyticus*, macrophage

## Abstract

Bovine mastitis, an inflammation of the mammary gland, is recognized as the most prevalent disease in dairy cattle, leading to significant economic losses due to the reduction in quality and yield of milk. While antibiotic treatment remains to be the primary control method but their use of antibiotics contributes to the emergence of antibiotic-resistant bacteria and possesses potential health risks to consumers. Enhancement of host immune responses represents a promising alternative strategy for combating pathogenic bacteria. This study aimed to evaluate the immunomodulatory potential of metabolites derived from lactic acid bacteria (LAB) in murine macrophage RAW264.7 cells infected with *Staphylococcus haemolyticus*, a causative agent of bovine mastitis. Notably, LAB-derived metabolites demonstrated significant, strain-specific immunostimulatory activity. A comparative metabolomic analysis confirmed that each strain possessed a unique metabolic profile, providing a chemical basis for these various responses. The most pronounced effects were observed with metabolites from the isolated strain *Enterococcus faecalis*, which markedly increased NO production. Furthermore, these metabolites upregulated the expression of key inflammatory genes, e.g., *iNOS*, *COX-2*, *TNF-α*, and *IL-6* and enhanced the protein levels of iNOS and COX-2. These findings suggest that LAB metabolites, particularly those from *E. faecalis*, may offer a novel therapeutic approach for enhancing immune defenses against mastitis-causing pathogens.

## Simple Summary

Bovine mastitis, a painful udder infection in dairy cows, is a major economic burden for farmers. Although antibiotics are commonly used, their overuse of the antibiotics leads to resistance and milk safety concerns. Natural compounds from beneficial lactic acid bacteria that might help cows fight *Staphylococcus haemolyticus*, a common mastitis pathogen, were investigated in this study. In lab tests using mouse immune cells, these compounds boosted defenses by increasing nitric oxide (a natural antibacterial agent) and activating inflammation-related immune signals. Metabolites from *Enterococcus faecalis* were especially effective. These results suggested that such natural compounds could become antibiotic-free treatments for mastitis. This approach would benefit dairy farmers by lowering costs, improvement of cow welfare, and providing consumers with safer milk by reducing antibiotic use. Therefore, the compounds from the beneficial bacteria have potential as a sustainable solution for mastitis control.

## 1. Introduction

Bovine mastitis is a prevalent and economically significant disease in dairy cattle, characterized by inflammation of the mammary gland that is primarily caused by bacterial infections [1,2]. Among the various pathogens associated with mastitis, *Staphylococcus haemolyticus* and other coagulase-negative staphylococci (CNS) have been increasingly implicated in both subclinical and clinical forms [3,4]. Notably, *S. haemolyticus* is recognized as an emerging opportunistic pathogen with strong biofilm-forming ability and multidrug resistance, which enables it to persist within the mammary gland and evade host immune clearance. Infected cows often exhibit chronic or recurrent mastitis, leading to reduced milk yield, altered milk composition, and significant economic losses in dairy production. Moreover, the bacteria contain transferable resistance genes, posing a potential threat to animal health and raising public health concerns due to the possible transmission of antimicrobial resistance between animals and humans [5,6]. Gram-positive bacterial infections often trigger a delayed or insufficient host immune response, which can exacerbate disease severity [7]. Conventional treatment of bovine mastitis mostly relies on antibiotics to eliminate the causative bacteria and mitigate mammary gland inflammation. While data from the National Institute for Research in Dairying (NIRD) indicate that antibiotics effectively reduce bacterial load and clinical symptoms, this approach has critical limitations, including the emergence of antibiotic resistance, milk residues, and high recurrence rates [8]. Moreover, the overuse of antibiotics in dairy farming raises environmental and public health concerns [4]. Given these challenges, strengthening the host immune response to enable rapid and effective pathogen clearance is essential for disease control. Immunomodulatory strategies might present a viable alternative, offering potential improvements in mastitis management while reducing dependence on antimicrobial therapy.

Probiotics, particularly lactic acid bacteria (LAB), have garnered considerable attention for their immunomodulatory potential due to their ability to modulate host immune responses [9,10]. LAB are commonly found in fermented foods and the gut microbiota. They are recognized for their ability to enhance both innate and adaptive immunity [11,12]. These bacteria stimulate the production of key cytokines, enhance the activity of macrophages and dendritic cells, and promote immune homeostasis. Importantly, LAB reduce inflammation by regulating the overproduction of pro-inflammatory cytokines while upregulating regulatory cytokines like IL-10 [13], thereby mitigating excessive immune activation [14]. This immunoregulatory capacity makes LAB as promising candidates for supporting immune function, particularly in the context of infections and inflammatory conditions. Given these properties, probiotic-based interventions, especially those involving LAB, are being explored for both prophylactic and therapeutic applications. Their potential of the metabolite to complement or reduce reliance on conventional antibiotic treatments highlights their growing importance in contemporary medical and nutritional approaches.

Despite the well-documented immunomodulatory effects of lactic acid bacteria (LAB), the specific interaction between LAB-derived metabolites and *Staphylococcus haemolyticus* in the context of immune regulation has not been systematically investigated and the significant knowledge remains unknown. To address this, the present study aims to evaluate the potential of LAB-derived metabolites to modulate immune responses in RAW264.7 macrophage cells following *S. haemolyticus* infection.

## 2. Materials and Methods

### 2.1. Reagents and Chemicals

De Man, Rogosa, and Sharpe (MRS) medium and brain heart infusion medium were purchased from HiMedia (Dindori, Nashik, India). Dulbecco’s Modified Eagle’s Medium (DMEM), fetal bovine serum, and penicillin-streptomycin were obtained from Gibco (Grand Island, NY, USA). 3-(4,5-Dimethylthiazol-2-yl)-2,5-diphenyltetrazolium bromide (MTT) was purchased from Bio Basic (Toronto, ON, Canada). Additionally, 2,2-diphenyl-1-picrylhydrazyl (DPPH), 2,2′-azino-bis (3-ethylbenzothiazoline-6-sulfonic acid) (ABTS), 6-hydroxy-2,5,7,8-tetramethylchroman-2-carboxylic acid (Trolox), and gallic acid monohydrate were acquired from Sigma-Aldrich (St. Louis, MO, USA). Anti-iNOS/NOS II polyclonal, anti-COX-2 polyclonal, and anti-β-actin monoclonal antibodies, as well as peroxidase-conjugated secondary antibody, were purchased from EMD Millipore Corporation (Temecula, CA, USA).

### 2.2. Bacterial Strain

This study utilized selected lactic acid bacteria (LAB) strains isolated from raw milk, including *Enterococcus faecalis* TCAN02 (GenBank accession: OM992233.1) and *Lactobacillus plantarum* AD73 (GenBank accession: OM807268.1). All isolates were precisely identified through 16S rRNA gene sequencing, with their sequences deposited in the National Center for Biotechnology Information (NCBI) database. Additionally, reference strains *L. plantarum* TISTR 2070 and *L. casei* TISTR 1340 were included in the study. The *Staphylococcus haemolyticus* strain, isolated from bovine mastitis cases, was generously provided by Prof. Dr. Witaya Suriyasathaporn from Faculty of Veterinary Medicine, Chiang Mai University, Thailand.

### 2.3. Preparation of LAB-Derived Metabolites

LAB-derived metabolites were isolated from culture supernatants of both isolated and reference LAB strains [15]. The bacterial cultures were grown in De Man, Rogosa, and Sharpe (MRS) broth at a concentration of 1.0 × 10^8^ CFU/mL (100 mL total volume) and incubated for 48 h at 37 °C under static conditions. Following incubation, the culture supernatant was separated by centrifugation at 3000× *g* for 10 min at 4 °C. The cell-free supernatant was then sterilized by passage through a 0.22 µm pore-size membrane filter. The filtered supernatant was concentrated by lyophilization at −50 °C under reduced pressure until complete dehydration was achieved. The metabolite powder was obtained and reconstituted in sterile ultrapure water to a final concentration of 200 mg/mL and stored at −80 °C until use.

### 2.4. Preparation of Inactivated S. haemolyticus Bacterial Cells

The inactivation of *S. haemolyticus* bacterial cells was performed according to the method described by Dehlink et al. (2007) [16] with modifications. Briefly, *S. haemolyticus* was cultured in 100 mL of brain heart infusion (BHI) broth for 24 h at 37 °C. Bacterial cells were harvested by centrifugation at 3000× *g* for 15 min at 4 °C and washed twice with phosphate-buffered saline (PBS). The cell pellet was then inactivated by incubation with 4% paraformaldehyde (PFA) for 2 h on ice. Complete inactivation was verified by plating the treated cells on BHI agar plates and incubating at 37 °C for 48 h, with observation of non-bacterial growth confirming successful inactivation.

### 2.5. Cell Culture

Murine macrophage RAW 264.7 cells (ATCC TIB-71) were maintained in Dulbecco’s Modified Eagle Medium (DMEM) supplemented with 10% (*v*/*v*) heat-inactivated fetal bovine serum (FBS) and 1% (*v*/*v*) penicillin-streptomycin antibiotics. The cells were cultured under standard conditions in a humidified incubator with 5% CO_2_ at 37 °C, with media changes performed every 2–3 days. Cells were routinely subcultured when 80–90% cell confluence was observed using cell scrapers for the further experiment.

### 2.6. Cell Viability

Cell viability was assessed by measuring mitochondrial dehydrogenase activity using the MTT (3-(4,5-dimethylthiazol-2-yl)-2,5-diphenyltetrazolium bromide) assay [17]. RAW 264.7 macrophages were seeded in 96-well plates at a density of 1.0 × 10^5^ cells/well and allowed to adhere for 24 h prior to treatment. Cells were then exposed to LAB-derived metabolite solutions (100 µL/well) at concentrations ranging from 11 to 20 mg/mL. Following 24 h of incubation, the culture medium was carefully aspirated and replaced with MTT solution. After 2–4 h of additional incubation at 37 °C, the formazan crystals were solubilized with 200 µL of dimethyl sulfoxide (DMSO) per well. Absorbance was measured using a microplate reader (DYNEX Technologies, Chantilly, VA, USA) at 540 nm with a reference wavelength of 630 nm. Cell viability was expressed as a percentage relative to untreated control cells (set as 100% viability).

### 2.7. Infection Studies with Inactivated S. haemolyticus

For bacterial challenge experiments, RAW 264.7 cells were co-cultured with *S. haemolyticus* (I/A) at multiplicity of infection (MOIs) ratios ranging from 1.56 to 50 for 24 h. Following infection, cells were gently washed twice with 100 µL phosphate-buffered saline (PBS) before assessing cell viability using the MTT assay as described.

### 2.8. Measurement of Nitric Oxide Production

Nitric oxide (NO) production was measured in RAW 264.7 murine macrophages using the Griess assay [18] to evaluate the effects of LAB-derived metabolites under both basal and *S. haemolyticus* (I/A)-stimulated conditions. Cells were seeded in 96-well plates at a density of 1.0 × 10^5^ cells/well and allowed to adhere for 24 h. The culture medium was then replaced with non-toxic concentrations of LAB-derived metabolites and incubated for an additional 24 h. Following incubation, 100 µL of culture supernatant was collected and mixed with an equal volume of Griess reagent (1% sulfanilamide in 5% phosphoric acid containing 0.1% N-(1-naphthyl) ethylenediamine dihydrochloride). After 15 min of incubation at room temperature in the dark, absorbance was measured at 550 nm using a microplate reader. Appropriate controls were included: LAB-derived metabolite solutions alone served as reference blanks to account for potential interference, while cells treated with 5 ng/mL lipopolysaccharide (LPS) provided a positive control (set as 100% NO production).

### 2.9. Morphological Analysis of Macrophage Activation

Morphological changes in RAW264.7 macrophages were assessed by Giemsa staining [19]. Cells were seeded in 24-well plates at a density of 5.0 × 10^5^ cells/well in complete DMEM (10% FBS, 1% penicillin/streptomycin) and allowed to adhere for 24 h at 37 °C in a 5% CO_2_ humidified incubator. Then, cells were treated with subtoxic concentrations of LAB-derived metabolites in combination with subtoxic doses of *S. haemolyticus*. Following 24 h of incubation under standard culture conditions, cells were washed twice with PBS, fixed with 4% paraformaldehyde, and stained with Giemsa solution. Stained cells were visualized using an inverted light microscope

### 2.10. Analysis of Inflammatory Gene Expression by Quantitative Real-Time PCR

Gene expression levels of *iNOS*, *COX-2*, *IL-6*, and *TNF-α* were analyzed using quantitative reverse transcription polymerase chain reaction (qRT-PCR). RAW 264.7 murine macrophages were seeded in 24-well plates at a density of 5.0 × 10^5^ cells/well and allowed to adhere for 24 h prior to treatment. Cells were then stimulated with LAB-derived metabolites (4, 8, and 16 mg/mL) in the presence of inactivated *S. haemolyticus* (I/A) at a subtoxic doses with multiplicity of infection (MOI) of 25, followed by incubation for 24 h under standard culture conditions (37 °C, 5% CO_2_, humidified atmosphere). Total RNA was isolated using TRIzol^®^ reagent (Invitrogen, Carlsbad, CA, USA) according to the manufacturer’s protocol. cDNA synthesis was performed using the ReverTra Ace^®^ qPCR RT Master Mix (TOYOBO, Osaka, Japan). Quantitative PCR amplification was carried out using the SensiFAST™ SYBR^®^ No-ROX Kit (BIOLINE, London, UK) on a real-time PCR system. The β-actin gene served as the endogenous reference control for normalization, with primer sequences provided in Table 1. Relative gene expression was calculated using the 2^(−ΔΔCt)^ method.

### 2.11. Immunoblotting Analysis of Protein Expression

Protein expression levels of iNOS and COX-2 were evaluated in RAW 264.7 murine macrophages by Western blot analysis. Cells were seeded in 12-well plates at a density of 1.0 × 10^6^ cells/well and allowed to adhere for 24 h prior to treatment with LAB-derived metabolites. Following 24 h of incubation under standard culture conditions (37 °C, 5% CO_2_), cells were lysed and total protein was extracted. Equal amounts of protein were separated by SDS-PAGE and transferred to PVDF membranes. Membranes were blocked with 5% non-fat dry milk in TBST (Tris-buffered saline with 0.1% Tween-20) for 1 h at room temperature, followed by overnight incubation at 4 °C with primary antibodies against iNOS (polyclonal, 1:1000), COX-2 (polyclonal, 1:1000), and β-actin (monoclonal, 1:1000). After washing, membranes were incubated for 1 h at room temperature with species-appropriate horseradish peroxidase (HRP)-conjugated secondary antibodies (goat anti-mouse or anti-rabbit IgG, 1:1000). Protein bands were visualized using Ultrascene Pico Chemiluminescent Substrate (Bio-helix, New Taipei City, Taiwan) and detected using the ImageQuant LAS 500 Chemiluminescent Imaging System (GE Healthcare, Boston, MA, USA). Band intensities were quantified using ImageJ software version 1.54p (NIH, Bethesda, MD, USA) and normalized to β-actin expression levels, with results expressed as fold-changes relative to untreated controls (set as 1.0).

### 2.12. DPPH Radical Scavenging Assay

The antioxidant capacity of LAB-derived metabolites was evaluated using the 2,2-diphenyl-1-picrylhydrazyl (DPPH) radical scavenging assay [24]. Briefly, serial dilutions of the metabolite samples were prepared and mixed with 0.1 mM DPPH solution. The reaction mixture was mixed and incubated in the dark at room temperature for 20 min to allow complete reaction. The absorbance was then measured at 517 nm using a microplate reader, with methanol serving as the blank control. The antioxidant capacity was quantified against a gallic acid standard curve and expressed as milligrams of gallic acid equivalents per gram of LAB-derived metabolites (mg GAE/g metabolites). All measurements were performed in triplicate.

### 2.13. ABTS Radical Scavenging Assay

The antioxidant capacity of LAB-derived metabolites was further evaluated using the 2,2′-azino-bis (3-ethylbenzothiazoline-6-sulfonic acid) (ABTS) radical cation decolorization assay [25]. The ABTS radical cation (ABTS•+) was generated by reacting 7 mM ABTS stock solution with 2.45 mM potassium persulfate (final concentration) in distilled water, followed by 12–16 h of incubation in the dark at room temperature. Prior to analysis, the ABTS•+ solution was diluted with phosphate buffer (pH 7.4) to an absorbance of 0.70 ± 0.02 at 734 nm. For the assay, LAB-derived metabolite samples (5 μL) were mixed with 195 μL of diluted ABTS•+ solution in a 96-well microplate and incubated for exactly 10 min at room temperature protected from light. Absorbance was then measured at 734 nm using a microplate reader. Trolox (6-hydroxy-2,5,7,8-tetramethylchroman-2-carboxylic acid) was used as an antioxidant standard, and results were expressed as mg of Trolox equivalents (TE) per gram of LAB-derived metabolites (mg TE/g metabolites).

### 2.14. Metabolomic Analysis Using LC-MS/MS

Metabolomic analysis was performed using an Agilent 6545XT AdvanceBio QTOF LC-MS system (Agilent Technologies, Santa Clara, California, USA). Chromatographic separation was achieved on a Poroshell 120 EC-C18 column (2.1 × 100 mm; 2.7 µm) maintained at 50 °C. The mobile phase consisted of 0.1% formic acid in water (A) and 0.1% formic acid in acetonitrile (B). A gradient elution was employed at a flow rate of 0.4 mL/min as follows: 0% B for 0.5 min, increased to 55% B at 10.5 min, increased to 75% B at 12.5 min, and further increased to 100% B at 14.0 min. This was held at 100% B for 3 min before re-equilibrating to 0% B at 17.5 min for a total run time of 20 min. The injection volume was 10 µL.

Mass spectrometry was performed in both positive and negative high-resolution modes. The ion source parameters were set as follows: drying gas temperature at 325 °C with a flow rate of 13 L/h, sheath gas temperature at 275 °C with a flow rate of 12 L/h, and nebulizer pressure at 45 psi. The capillary voltage was set to 4000 V in positive mode and 3000 V in negative mode. Data were acquired in MS1 mode over a mass range of 40–1700 *m*/*z* and in MS2 mode over a range of 25–1000 *m*/*z*, at an acquisition rate of 3.35 spectra/s. The collision energy was set to 20 eV for positive mode and 10 eV for negative mode. Up to 10 precursors per cycle were selected for fragmentation with a precursor threshold of 5000 counts. Continuous internal calibration was performed using reference masses at m/z 121.0509 and 922.0098 in positive mode, and m/z 112.9856 and 1033.9881 in negative mode.

### 2.15. Statistical Analysis

All experimental data were analyzed using GraphPad Prism version 10 (GraphPad Software, Inc., La Jolla, CA, USA). Results are presented as mean ± standard deviation (SD) of at least three independent experiments, each performed in triplicate. For comparisons between two groups, statistical significance was determined using unpaired two-tailed *t*-test. Multiple group comparisons were analyzed by one-way analysis of variance (ANOVA) followed by Tukey’s post hoc test. In all analyses, a *p*-value < 0.05 was considered statistically significant.

## 3. Results

### 3.1. Cytotoxicity Evaluation of LAB-Derived Metabolites on RAW264.7 Murine Macrophages

**Figure 1:**
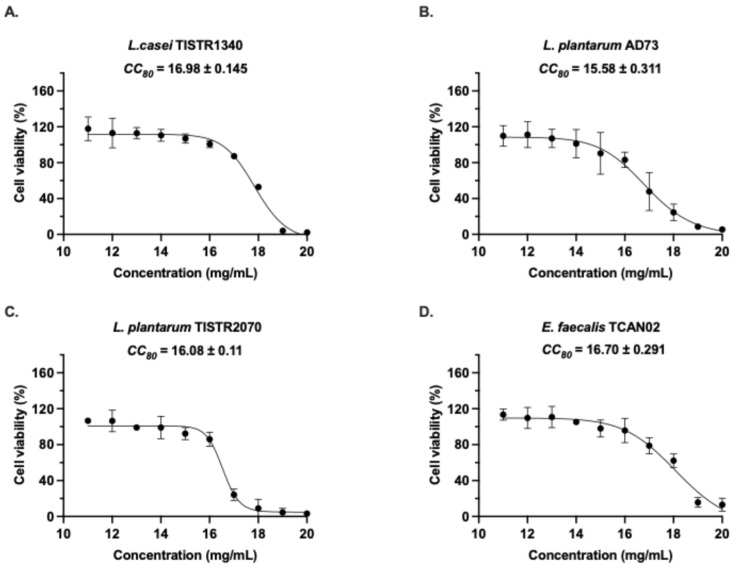
Cytotoxic effects of LAB-derived metabolites on RAW264.7 macrophage viability. Cells were treated for 24 h with increasing concentrations (11–20 mg/mL) of metabolites from (**A**) *L. casei* TISTR1340, (**B**) *L. plantarum* AD73, (**C**) *L. plantarum* TISTR2070, and (**D**) *Enterococcus faecalis* TCAN02. Cell viability was assessed by MTT assay and expressed as percentage relative to untreated controls (dotted line at 100%). Data represent mean ± SD of three independent experiments *(n* = 3), each performed in triplicate.

The cytotoxicity of LAB-derived metabolites from four bacterial strains were systematically evaluated in RAW264.7 murine macrophages using the MTT assay (Figure 1). Following 24 h exposure, all treatment demonstrated dose-dependent effects on cell viability, though with distinct strain-specific patterns. *L. casei* TISTR1340 metabolites exhibited cytotoxicity, with a CC_80_ of 16.98 ± 0.145 mg/mL, suggesting relatively low toxicity that might be high biocompatibility within the tested concentration range. *E. faecalis* TCAN02 metabolites induced the cytotoxic response at a CC_80_ of 16.70 ± 0.291 mg/mL. Similarly, *L. plantarum* TISTR2070 showed comparable effects at CC_80_ of 16.08 ± 0.11 mg/mL. Notably, *L. plantarum* AD73 displayed significantly higher cytotoxic potential, as evidenced by its lower CC_80_ of 15.58 ± 0.311 mg/mL. These findings demonstrated that while all tested metabolites affect macrophage viability in a concentration-dependent manner, the magnitude of cytotoxicity varies substantially between strains, with *L. plantarum* AD73 emerging as the most cytotoxic and *L. casei* TISTR1340 as the lowest toxicity.

### 3.2. Cytotoxicity Evaluation of Inactivated S. haemolyticus (I/A) on RAW264.7 Murine Macrophages

**Figure 2:**
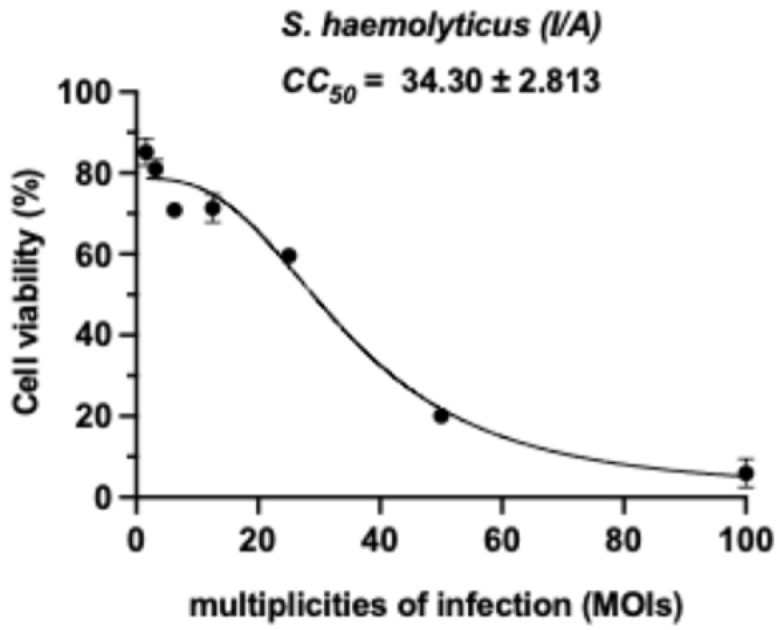
Cell viability of RAW264.7 macrophages after 24 h exposure to inactivated *S. haemolyticus* at varying MOIs. Dose-dependent cytotoxicity of inactivated *S. haemolyticus* in RAW264.7 macrophages. Cells were exposed to increasing multiplicities of infection (MOI: 1.56–100) of inactivated *S. haemolyticus* for 24 h, with viability assessed by MTT assay. Data represent mean ± SD of three independent experiments (n = 3), each performed in triplicate. Dotted line indicates cell viability threshold.

The cytotoxic effects of inactivated *S. haemolyticus* (I/A) on RAW264.7 murine macrophages were evaluated across a range of multiplicities of infection (MOI). As shown in Figure 2, macrophage viability was maintained at ≥80% at MOIs up to 12.5. Notably, cell viability remained above 50% at an MOI of 25, demonstrating the dose-dependent nature of the bacterial challenge, indicating a relatively safe concentration range for subsequent experiments.

### 3.3. Nitric Oxide (NO) Production on RAW264.7 Murine Macrophages

**Figure 3:**
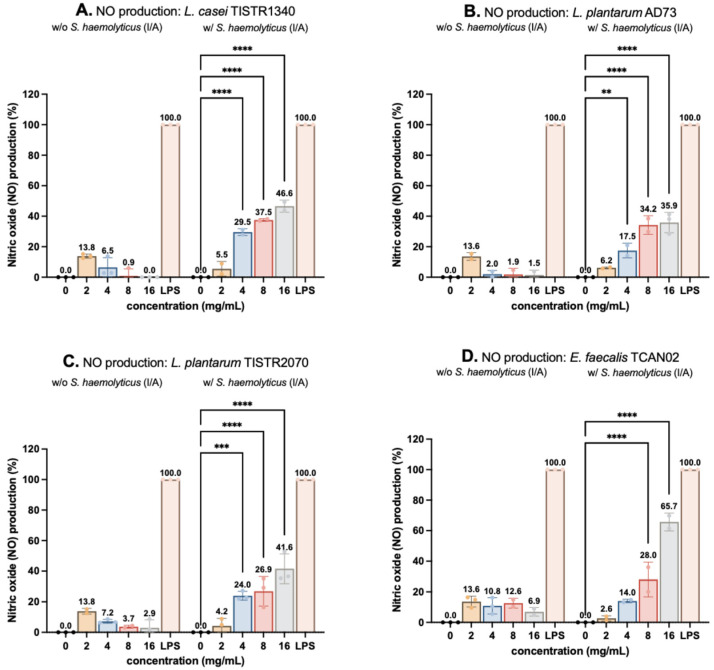
Modulation of nitric oxide (NO) production in RAW264.7 macrophages by LAB-derived metabolites. Cells were treated for 24 h with metabolites (2–16 mg/mL) from (**A**) *Lactobacillus casei* TISTR1340, (**B**) *L. plantarum* AD73, (**C**) *L. plantarum* TISTR2070, or (**D**) *Enterococcus faecalis* TCAN02, either alone or in combination with inactivated *S. haemolyticus*. LPS (5 ng/mL) served as a positive control (100% response). Data represent mean ± SD of triplicate experiments. ** indicates *p* < 0.005; *** indicates *p* < 0.0005; and **** indicates *p* < 0.0001.

**Figure 4:**
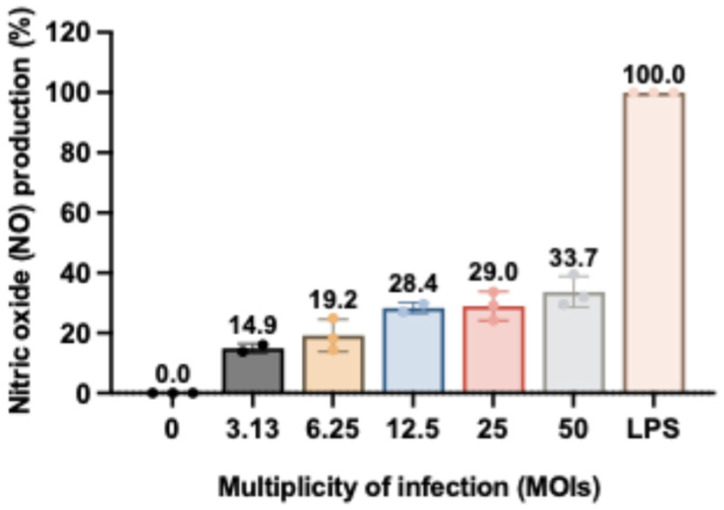
Immunostimulatory effect of inactivated *S. haemolyticus* on nitric oxide (NO) production in RAW264.7 murine macrophages. Cells were treated with inactivated *S. haemolyticus* (I/A) for 24 h, and NO concentration in the culture supernatant was measured. LPS (5 ng/mL) served as a positive control (100% response). Data represent mean ± SD of triplicate experiments.

The immunomodulatory effects of LAB-derived metabolites were assessed by measuring NO production in RAW264.7 macrophages using the Griess assay. Cells were treated with subtoxic concentrations of metabolites (2–16 mg/mL) in the presence or absence of *S. haemolyticus* (I/A), with LPS (5 ng/mL) serving as a positive control (100% NO production). While treatment alone induced minimal NO release, co-stimulation with *S. haemolyticus* (I/A) triggered dose-dependent increases in NO production (Figure 3D). *Enterococcus faecalis* TCAN02 metabolites elicited the strongest response (65.68% of LPS-induced NO), followed by *Lactobacillus casei* TISTR1340, *L. plantarum* TISTR2070, and *L. plantarum* AD73 metabolites. Notably, *S. haemolyticus* alone at MOI 50 induced moderate NO production (33.68%), confirming its immunostimulatory potential (Figure 4). These results demonstrated that LAB metabolites synergized with bacterial challenge to enhance macrophage activation, with strain-specific efficacy.

### 3.4. LAB-Derived Metabolites Induce Distinct Morphological Activation in S. haemolyticus (I/A)-Challenged RAW264.7 Murine Macrophages

**Figure 5:**
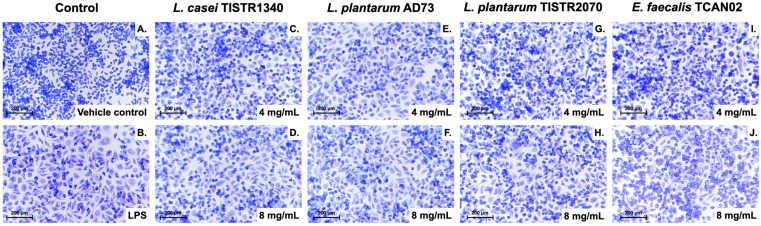
Morphological activation of RAW264.7 macrophages induced by LAB-derived metabolites during *S. haemolyticus* (I/A) challenge. Giemsa-stained cells showing: (**A**) Resting morphology in unstimulated controls, (**B**) Classical activation with LPS (positive control), and (**C**–**J**) Dose-dependent morphological changes (4–8 mg/mL) with *L. casei* TISTR1340 (**C**,**D**), *L. plantarum* AD73 (**E**,**F**), *L. plantarum* TISTR2070 (**G**,**H**), and *E. faecalis* TCAN02 (**I**,**J**) metabolites during bacterial co-stimulation.

Morphological changes in RAW264.7 macrophages under various co-stimulation conditions were assessed by Giemsa staining, as depicted in Figure 5. The results revealed that LAB-derived metabolites induced characteristic activation-associated morphological changes in *S. haemolyticus*-challenged RAW264.7 macrophages. Untreated control cells maintained a flattened, adherent morphology with extensive cytoplasmic projections. However, co-treatment of macrophages with LAB metabolites and *S. haemolyticus* (I/A) exhibited dose-dependent alterations, including cellular rounding, membrane ruffling, and reduced substrate adhesion, which is hallmarks of macrophage activation. Notably, *L. plantarum* AD73 and *E. faecalis* TCAN02 metabolites at 8 mg/mL elicited morphological changes comparable to LPS-stimulated positive controls, suggesting immunostimulatory capacity.

**Figure 6:**
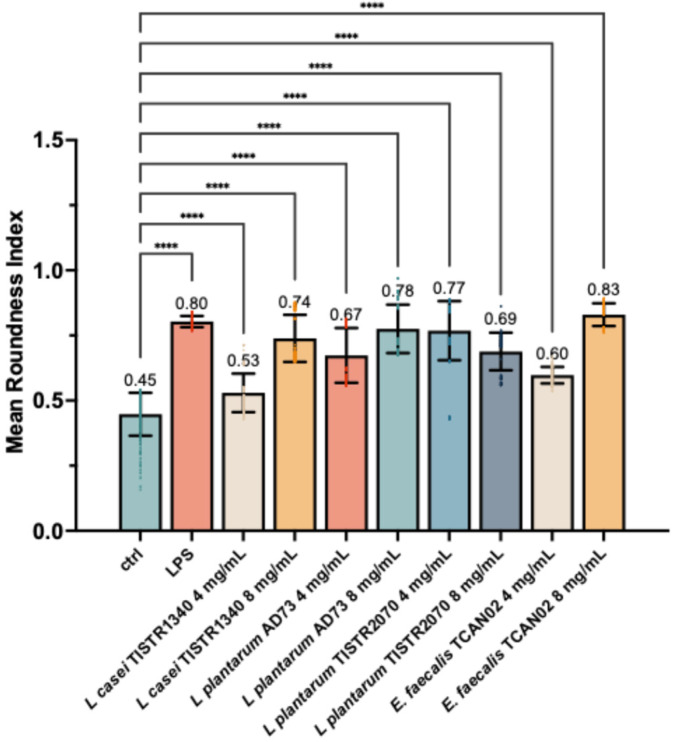
Quantitative analysis of macrophage activation by cell circularity. The circularity of cells from representative images was quantified using ImageJ software. Data are presented as mean ± SD (n = 3 independent experiments, with >100 cells analyzed per group per experiment). Statistical significance relative to the vehicle control group was determined by One-way ANOVA followed by Tukey’s post hoc test. **** indicates *p* < 0.0001.

For quantitative validation for these morphological observations, cells were measured the cell circularity metric using ImageJ software (Figure 6). The analysis confirmed that unstimulated cells maintained a low circularity score, consistent with their flattened, adherent morphology.

In contrast, the LPS-stimulated positive control showed a significant increase in cell circularity, indicative of cellular rounding. Similarly, all LAB-derived metabolites induced a statistically significant, dose-dependent increase in cell circularity. Notably, treatment with 8 mg/mL of metabolites from *L. plantarum* AD73 and *E. faecalis* TCAN02 resulted in the most pronounced cellular rounding, achieving activation levels comparable to the LPS control. These quantitative data statistically confirm that LAB-derived metabolites promote significant morphological activation in *S. haemolyticus*-challenged macrophages.

### 3.5. Modulation of Pro-Inflammatory Cytokine Gene Expression on RAW264.7 Murine Macrophages

**Figure 7:**
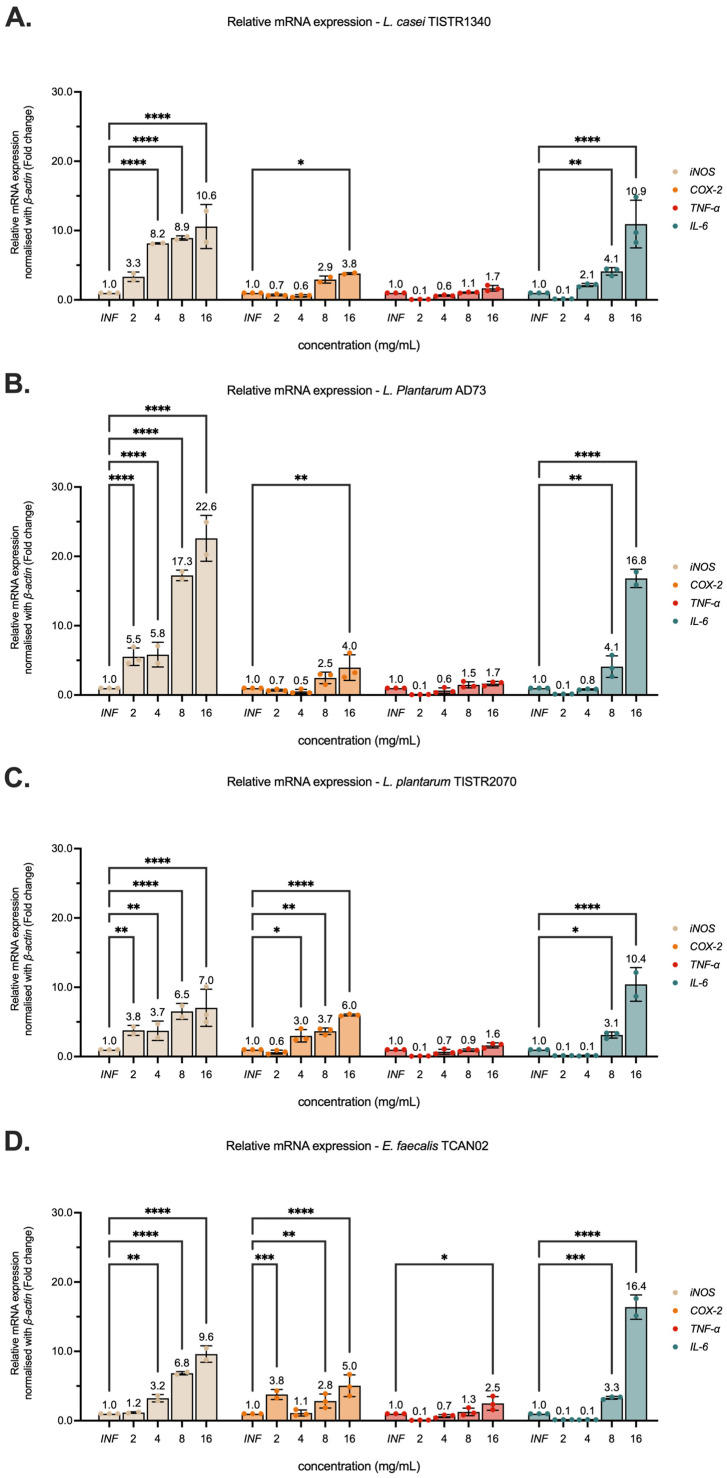
Modulation of pro-inflammatory cytokine gene expression by LAB-derived metabolites in RAW264.7 macrophages. Cells were treated for 24 h with metabolites (2–16 mg/mL) from (**A**) *Lactobacillus casei* TISTR1340, (**B**) *L. plantarum* AD73, (**C**) *L. plantarum* TISTR2070, or (**D**) *Enterococcus faecalis* TCAN02, with *S. haemolyticus* (I/A). Gene expression levels of TNF-α, IL-6, iNOS, and COX-2 were quantified by qRT-PCR and normalized to β-actin. Data represent mean ± SD of triplicate experiments. * indicates *p* < 0.05; ** indicates *p* < 0.005; *** indicates *p* < 0.0005; and **** indicates *p* < 0.0001.

qRT-PCR analysis demonstrated that LAB-derived metabolites (2–16 mg/mL) significantly enhanced the mRNA expression of pro-inflammatory mediators (*iNOS*, *COX-2*, *TNF-α*, and *IL-6*) in RAW 264.7 macrophages infected with *S. haemolyticus* (I/A) (Figure 7). Notably, *L. plantarum* AD73 metabolites exhibited the strongest immunostimulatory effects, inducing *iNOS* and *IL-6* expression by 22.59-fold and 16.82-fold, respectively (*p* < 0.01 versus untreated controls). In contrast, *L. plantarum* TISTR2070 metabolites showed preferential upregulation of *COX-2* at a value of 18.43 ± 1.1-fold, *p* < 0.05. While all tested LAB strains (including *L. casei* TISTR1340 and *E. faecalis* TCAN02) increased cytokine expression in a dose-dependent manner. The observation of strain-specific patterns of immunostimulatory effects suggests distinct immunomodulatory mechanisms. These findings highlight the potential of LAB-derived metabolites to enhance macrophage inflammatory responses during bacterial challenge. The metabolites from *L. plantarum* AD73 demonstrated particularly potent activity.

### 3.6. LAB-Derived Metabolites Potently Upregulate Inflammatory Mediators on RAW264.7 Murine Macrophages Exposed to S. haemolyticus

**Figure 8:**
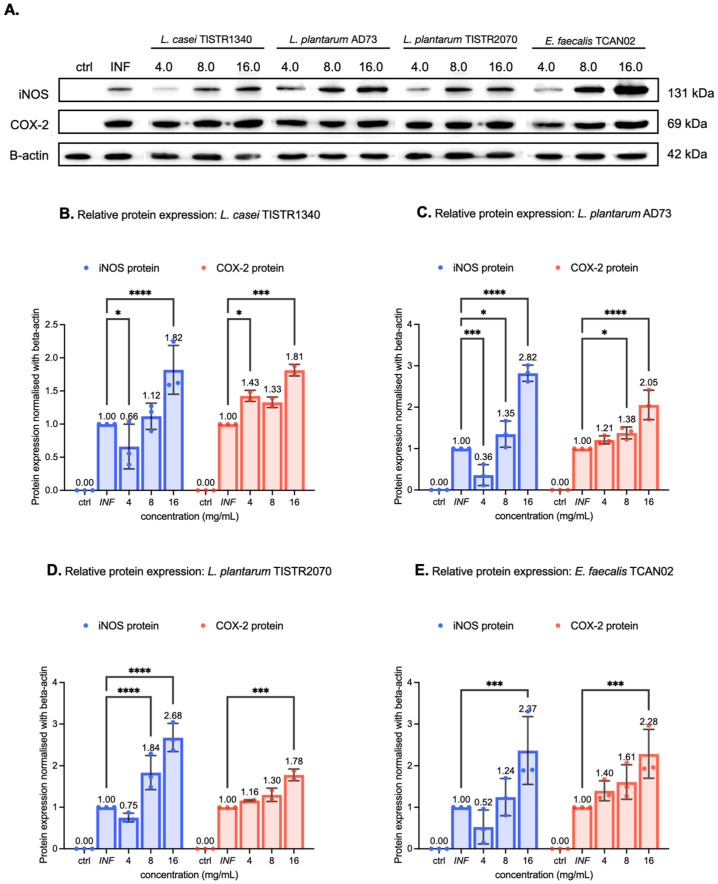
Modulation of iNOS and COX-2 protein expression by LAB-derived metabolites in *S. haemolyticus*-challenged macrophages. (**A**) Representative Western blots show dose-dependent up-regulation of iNOS and COX-2 protein levels after 24 h co-treatment with LAB metabolites. Relative protein expressions of iNOS and COX-2 were demonstrated after co-treatment with (**B**) *Lactobacillus casei* TISTR1340, (**C**) *L. plantarum* AD73, (**D**) *L. plantarum* TISTR2070, or (**E**) Entero-coccus faecalis TCAN02 metabolites (4.0–16.0 mg/mL). Protein levels were analyzed by immunoblotting with β-actin as loading control. Densitometric quantification represents mean ± SD of three independent experiments. * indicates *p* < 0.05; *** indicates *p* < 0.0005; and **** indicates *p* < 0.0001.

Immunoblotting analysis demonstrated that LAB-derived metabolites significantly enhanced the expression of inflammatory proteins iNOS and COX-2 in RAW264.7 macrophages challenged with *S. haemolyticus* (I/A). All treatments exhibited dose-dependent effects (4–16 mg/mL), with particularly responses observed at the highest concentration (16 mg/mL) (Figure 8 and Figures S1–S3). Notably, metabolites from *L. plantarum* TISTR2070 increased iNOS expression by 50% at 8 mg/mL, while both *L. plantarum* TISTR2070 and *L. casei* TISTR1340 metabolites enhanced iNOS levels more than twice (>100% increase) and elevated COX-2 by more than 50% at 16 mg/mL. Among isolated strains, *L. plantarum* AD73 metabolites (16 mg/mL) upregulated iNOS (>50%) and COX-2 (100%) expression, while *E. faecalis* TCAN02 metabolites showed the most potent immunomodulatory effects, increasing both inflammatory markers by over 100%. These findings demonstrate that LAB metabolites can substantially amplify macrophage inflammatory responses to bacterial challenge, with the magnitude of effect varying significantly between different bacterial strains. The strong upregulation of iNOS and COX-2 suggests LAB metabolites may enhance antimicrobial defenses through potent activation of inflammatory pathways.

### 3.7. Antioxidant Activity of LAB-Derived Metabolites

The antioxidant potential of LAB-derived metabolites was evaluated using DPPH and ABTS radical scavenging assays, revealing distinct strain-specific activities (Table 2). DPPH assay results demonstrated moderate antioxidant capacity across all strains ranging of 1.25–1.84 mg GAE/g sample. LAB-derived metabolites from *L. casei* TISTR1340 showed the highest capacity at a value of 1.84 ± 0.21 mg GAE/g sample. In contrast, ABTS assay revealed higher antioxidant capacity ranging from 7.44–14.97 mg TE/g sample. LAB-derived metabolites from *L. plantarum* AD73 exhibited the most potent effects at a value of 14.97 ± 2.52 mg TE/g sample.

### 3.8. Multivariate Analysis Reveals Distinct Metabolic Profiles Between Bacterial Species

Untargeted LC-MS metabolomics analysis revealed that the four bacterial species induced distinct and highly significant metabolic shifts in the culture medium, as evidenced by multivariate statistical analysis of both positive and negative ionization mode. The results from the supernatants of *E. faecalis* TCAN02 (Group a), *L. casei* TISTR1340 (Group b), *L. plantarum* AD73 (Group c), and *L. plantarum* TISTR2070 (Group d) were compared against each other.

**Figure 9:**
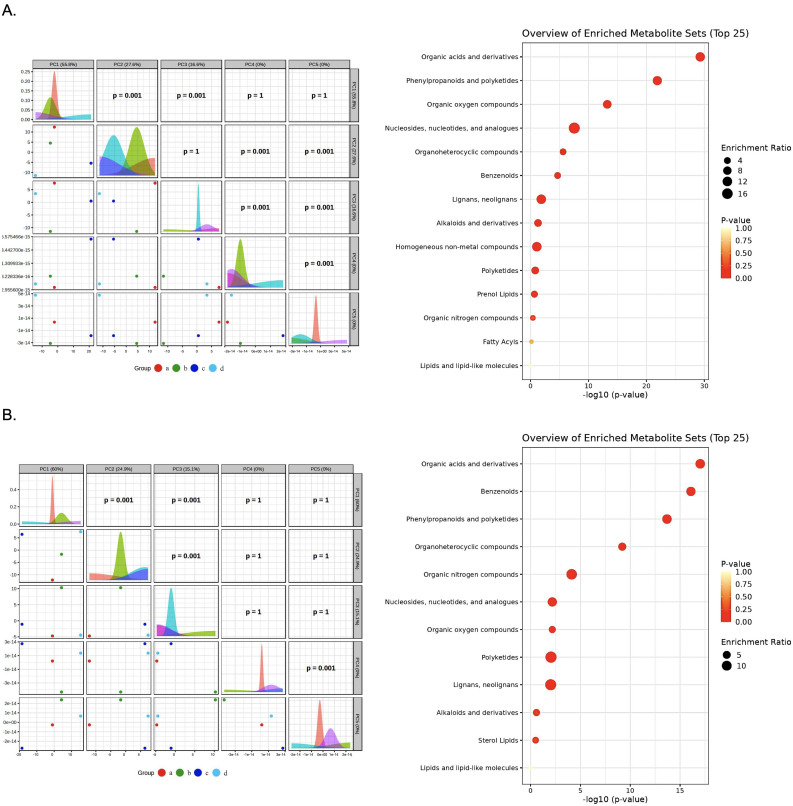
Comparative metabolomic profiling of Lactic Acid Bacteria (LAB)-derived metabolites. Analysis was performed by LC-MS in (**A**) negative ion mode and (**B**) positive ion mode. Principal Component Analysis (PCA) correlation matrix showing the metabolic separation between the four LAB strains including *E. faecalis* (a), *L. casei* (b), *L. paracasei* (c), and *L. plantarum* (d). Metabolite Set Enrichment Analysis (MSEA) dot plot of the top 25 enriched metabolite sets, ranked by statistical significance (−log10 (*p*-value)). The dot size represents the enrichment ratio, and the color indicates the *p*-value.

Principal Component Analysis (PCA) demonstrated a clear and consistent segregation of the samples into two primary clusters (Figure 9). This separation was remarkably robust, appearing in both negative and positive ionization modes. The first cluster was composed exclusively of *E. faecalis* TCAN02 (a) and *L. casei* TISTR1340 (b), indicating a high degree of metabolic similarity between them. The second, more dispersed cluster, contained *L. plantarum* AD73 (c) and *L. plantarum* TISTR2070 (d). This primary separation was driven by the first principal component (PC1) in both modes, which accounted for over 50% of the total variance, highlighting a major metabolic divergence between the (a, b) and (c, d) groups. The primary axis of variation, PC1, which accounted for a majority of the total variance (58.8% in negative and 60.0% in positive mode). The second principal component (PC2) further resolved the subtle differences between Groups a, b, and c, which formed a tighter cluster, indicating a higher degree of metabolic similarity among them. The statistical significance of these groupings was confirmed with *p*-values of 0.001 for the key component comparison.

### 3.9. Hierarchical Clustering and Identification of Metabolites

**Figure 10:**
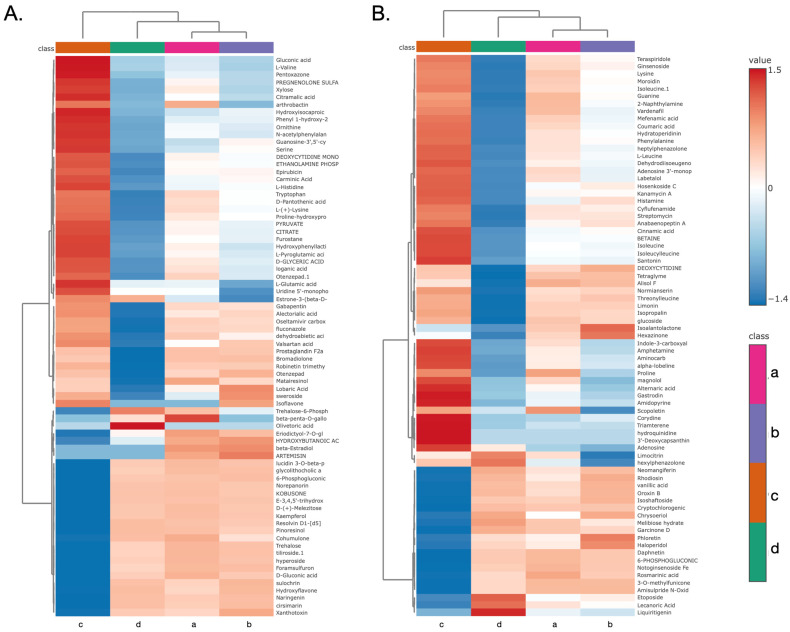
Heatmap and hierarchical clustering of significant metabolites identified in (**A**) negative ion mode and (**B**) positive ion mode. Columns correspond to the four LAB strains including *E. faecalis* (a), *L. casei* (b), *L. paracasei* (c), and *L. plantarum* (d), and rows correspond to individual metabolites. The relative abundance of metabolites is normalized (z-score) and visualized with a color scale where red indicates high abundance and blue indicates low abundance. The top dendrogram illustrates the similarity between the metabolic profiles of the strains, while the side dendrogram showed groups metabolites with similar expression patterns.

To investigate the specific molecular features driving these separations, hierarchical clustering analysis was performed on the top 75 differential metabolites. The resulting heatmaps and sample dendrograms explicitly corroborated the PCA findings, with the primary branch of the dendrogram separating Group C from a larger cluster containing Groups A, B, and D. Additionally, there is very similarity between Group A and B (Figure 10 and Table 3).

The metabolomic profiling conducted under both negative (Figure 10A) and positive (Figure 10B) ionization modes revealed distinct metabolic signatures among the four sample groups. Group D was characterized by a pronounced upregulation of metabolites within the upper cluster in both modes—for instance, olivetoric acid in the negative mode, and limocitrin and ginsenoside in the positive mode. In contrast, Group A exhibited a higher relative abundance of specific metabolites, including matairesinol in the negative mode, and rosmarinic acid and scopoletin in the positive mode. Group C presented a distinct metabolic profile, marked by elevated levels of glutamic acid and gluconic acid in the negative mode, and gastrodin and cinnamic acid in the positive mode. Notably, Group B displayed a unique metabolic phenotype, characterized by the presence of lobaric acid and sweroside in the negative mode, and phloretin in the positive mode.

### 3.10. Enrichment Analysis Define

To interpret these molecular changes into a broader biochemical context, metabolite set enrichment analysis (MSEA) was conducted on the significantly altered features. The analysis of both negative (Figure 9A) and positive (Figure 9B) mode data consistently identified organic acids and derivatives as the most significantly enriched metabolite set. Furthermore, significant enrichment was also observed for phenylpropanoids and polyketides and benzenoids, which aligned with the observed differential abundance of aromatic amino acids. This consistent enrichment across both analytical modes indicates that the metabolic differences between the experimental groups are fundamentally rooted in the differential regulation of central carbon and secondary metabolic pathways involving these key chemical classes.

## 4. Discussion

Bovine mastitis represents a globally prevalent disease with significant economic consequences for dairy production [26]. This condition, primarily caused by bacterial infections of the udder, substantially impacts milk production, increases veterinary costs, and raises concerns about antibiotic residue contamination in milk [27]. Severe cases may result in mortality of affected animals [28]. The causative pathogens are categorized as either contagious or environmental agent. Notable contagious pathogens include *Staphylococcus aureus*, *Streptococcus agalactiae*, and *S. haemolyticus*, which spread during lactation and exacerbate disease transmission [29]. While antibiotic therapy remains the primary treatment approach, its use presents limitations including the development of antibiotic-resistant bacteria and potential host toxicity [30]. Although antibacterial activity of probiotic metabolites has been reported [31], as well as their influence on immune responses in colorectal adenocarcinoma [32]. There are no reports description their immunomodulatory effects on macrophage cells, particularly in the context of pathogenic bacteria-induced bovine mastitis infection. These challenges highlight the importance of enhancing host immune responses as an alternative strategy for mastitis control.

The immune system is an integrated defense network comprising specialized organs, cells, and molecules that safeguard the host while maintaining homeostasis [31]. Macrophages are pivotal effectors of this system, functioning as sentinels in innate immunity, mediators of antigen presentation, and regulators of cytokine-driven responses. By coordinating pathogen clearance and immune modulation, they play a critical role in both host defense and immune balance [32]. In recent years, probiotics have gained considerable attention as potential immunomodulatory agents, and their mechanisms of action have been increasingly explored. One of their most recognized functions is the regulation of host immune responses [33]. Moreover, accumulating evidence indicates that certain probiotic strains exert strain-specific immunomodulatory effects on host immunity and immune cell function, partly through interactions with Toll-like receptors (TLRs) that activate interferon (IFN) signaling pathways [34].

**Figure 11:**
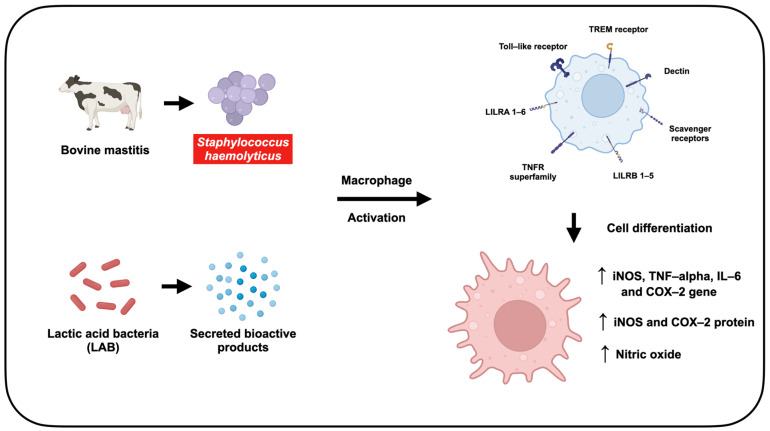
LAB-derived metabolites activate immune responses in RAW264.7 macrophages by promoting cell differentiation and stimulating nitric oxide production—a key host defense mechanism against *Staphylococcus haemolyticus* infection in bovine mastitis.

This study investigated the immune-stimulating potential of LAB-derived metabolites in *S. haemolyticus*-exposed RAW264.7 murine macrophages (Figure 11). RAW264.7 macrophage cell line was used as a standard in vitro model in many studies. The key pathways for investigation including nitric oxide (NO) production via iNOS and the expression of pro-inflammatory mediators such as COX-2, TNF-α, and IL-6 were highly conserved across mammalian species, including mice and cattle [35]. It is widely accepted and well characterized for study of fundamental macrophage immunology [36]. Moreover, the use of macrophage cell line ensures high reproducibility and consistency for the initial screening study. However, bovine macrophage should be used for further studied in the future.

The effect of LAB-derived metabolite on the murine macrophage RAW264.7 cell was determined through mitochondrial activity by MTT assay. The assay quantified mitochondrial activity through conversion of MTT [3-(4,5-dimethylthiazol-2-yl)-2,5-diphenyltetrazolium bromide] to insoluble formazan by mitochondrial reductase [37]. Results demonstrated strain-dependent effects on macrophage viability, with *L. plantarum* TISTR 2070 and AD73 metabolites exhibiting greater potency with higher CC_80_ compared to *L. casei* TISTR 1340 and *E. faecalis* TCAN02 metabolites. Co-incubation studies with *S. haemolyticus* (I/A) revealed maintained macrophage viability (>50%) at MOI 25, indicating low responses under bacterial challenge conditions. The immunomodulatory effects of LAB-derived metabolites were further evaluated through nitric oxide (NO) production in RAW264.7 macrophages. NO functions as a key signaling molecule involved in diverse cellular processes, including inflammatory responses and immune regulation [34]. Previous research has established its role in macrophage immune function, antiviral activity, and anti-cancer effects [37,38]. These properties make NO production a relevant biomarker for assessing immune modulation by LAB-derived metabolites.

NO production in RAW264.7 macrophages was quantified using the Griess assay, where nitrite reacts with sulphanilamide under acidic conditions to form a diazonium salt that couples with N-(1-naphthyl)ethylenediamine (NED), producing a colored azo dye [39]. At MOI 25, *S. haemolyticus* (I/A) alone induced 28.99% NO production relative to LPS controls that induced 100% NO production. However, co-treatment of *S. haemolyticus* (I/A) with LAB-derived metabolites, particularly from *E. faecalis* TCAN02, significantly enhanced NO production to 65.68%. Notably, LAB metabolites alone did not stimulate NO production in unchallenged macrophages, indicating their immune-activating effects specifically require bacterial co-stimulation. These findings demonstrate that LAB-derived metabolites potentiate macrophage immune responses primarily during pathogenic bacterial challenge rather than a non-specific stimulation.

Additionally, the expression levels of *iNOS*, *COX-2*, *TNF-α*, and *IL-6* genes were evaluated by qRT-PCR using β-actin as a reference gene. LAB-derived metabolites upregulated the expression of all examined genes, with particularly pronounced effects on *iNOS* and *IL-6*. TNF-α promotes inflammatory cell recruitment through regulation of leukocyte and vascular adhesion molecule expression [40], while IL-6 has been shown to mitigate tissue inflammation in hypersensitivity pneumonitis and reduce lung injury from oxygen toxicity and endotoxin exposure [41,42]. These observations are consistent with the potential role of LAB-derived metabolites in enhancing immune responses against bacterial pathogens. Immunoblotting analysis was performed to confirm the immunostimulatory activity of LAB-derived metabolites through evaluation of iNOS and COX-2 protein expression. The results demonstrated dose-dependent upregulation of both iNOS and COX-2 proteins following treatment with LAB-derived metabolites. In contrast, macrophages exposed to *S. haemolyticus* (I/A) showed lower expression levels of these proteins, indicating a diminished immune response in the absence of LAB metabolites. Interestingly, treatment with 2.0 mg/mL of LAB-derived metabolites from *E. faecalis* TCAN02 resulted in a higher COX-2 mRNA expression level compared with 4.0 and 8.0 mg/mL of LAB-derived metabolites. However, the corresponding protein expression exhibited a clear dose-dependent increase. This apparent discrepancy between mRNA and protein levels may be attributed to several biological factors. Both mRNA and protein were measured at the 24 h time point in order to determine the levels of COX-2 transcription and translation. Thus, the elevated COX-2 mRNA expression observed when treatment with 2.0 mg/mL of LAB-derived metabolites may represent a transient transcription occurring around 24 h. However, when treatment at the higher concentrations of LAB-derived metabolites such as 8.0 mg/mL, the COX-2 mRNA level may occur earlier and decline by the time of measurement. Moreover, COX-2 protein accumulation is influenced by both synthesis and degradation processes. The dose-dependent increase in COX-2 protein detected by Western blot suggests that higher concentrations of *E. faecalis*-derived metabolites may enhance translational efficiency or stabilize the COX-2 protein, thereby reducing its degradation [41,42].

This study of LAB-derived metabolites revealed distinct profiles among different bacterial strains, leading to different immunomodulatory capacity. For more understanding the chemical profile of this variation, the comparative metabolic analysis was performed using LC-MS/MS. The results clearly demonstrated that the four LAB strains possessed unique and distinct metabolic profiles, which were directly responsible for their differential biological activities. The Principal Component Analysis (PCA) confirmed that the metabolic fingerprints of the four strains were statistically different. The clear separation of the groups in both positive and negative ion modes indicated that each strain produced a unique combination of metabolites. This fundamental of chemical dissimilarity explained why they elicited variation in responses in the macrophage. The metabolite set enrichment analysis (MSEA) further identified the major classes of compounds that defined these profiles. Across all strains, organic acids and derivatives, benzenoids, phenylpropanoids and polyketides were among the most significantly enriched classes. This is a critical finding, as these categories contain a vast number of known bioactive molecules, including phenolic acids and flavonoids, which are well-documented for their immunomodulatory effects.

Notably, several key compounds were identified in the LAB-derived metabolite profiles, including matairesinol from *E. faecalis* and cinnamic acid from *L. plantarum*. While the present study evaluated the effects of the whole metabolite mixtures, it is possible that these individual components are the primary contributors to the observed immunomodulatory responses [43,44]. Therefore, these compounds should be isolated and analyzed in order to determine whether individual compound can stimulate the immune response or synergistic interaction among multiple metabolites are required. The future investigations would provide more precise understanding of the molecular mechanisms underlying LAB-mediated immune modulation.

The potent ability of *E. faecalis* TCAN02 to induce nitric oxide (NO) production is likely driven by its unique metabolic signature. Matairesinol, identified in this strain, has been reported to promote M1 macrophage polarization in THP-1 cells [45]. Additionally, previous study showed effect of rosmarinic acid to enhance immune responses in ovalbumin-induced intestinal allergy mouse models [46], that might further contribute to this effect. Similarly, the strong upregulation of *iNOS* and *IL-6* gene expression observed in response to *L. plantarum* AD73 metabolites can be attributed to its distinct chemical profile, which exhibits comparatively high levels of glutamic acid, gluconic acid, gastrodin, and cinnamic acid relative to the other strains. With respect to antioxidant activity, the metabolic profiles also help to explain the divergent results observed in the antioxidant assays. The superior ABTS radical scavenging activity of *L. plantarum* AD73 correlates with the high abundance of specific phenolic compounds. In contrast, the enhanced DPPH radical scavenging capacity of *L. casei* TISTR1340 metabolites may be associated with its production of distinct organic acids or other specialized small molecular metabolites.

These findings demonstrate that LAB-derived metabolites induce immune responses in murine macrophages. When macrophages infected with *S. haemolyticus* (I/A) were treated with LAB-derived metabolites, NO production increased significantly. The expression levels of inflammatory mediator genes including inducible nitric oxide synthase (iNOS), cyclooxygenase-2 (COX-2), tumor necrosis factor-alpha (TNF-α), and interleukin-6 (IL-6)—were elevated, along with iNOS and COX-2 protein levels. LAB-derived metabolites activated the host immune response specifically in the presence of pathogenic bacteria, as no induction of NO production or upregulation of inflammatory genes/proteins occurred in uninfected macrophages. This pathogen-dependent activation highlights the therapeutic potential of LAB-derived metabolites for bacterial infections.

On the other hand, it is important to acknowledge certain limitations of the present study. A primary methodological limitation is the number of replicates used for our in vitro assays. The experiments were consistently performed with three independent replicates (*n* = 3). While this is a common practice for initial screening studies and our data yielded statistically significant trends, this relatively small sample size may impact the overall statistical power and broad reproducibility of the results. Therefore, the future studies should validate these promising findings using a larger number of data and confirm the reliability of these metabolites before proceeding to primary cell or in vivo models. Moreover, antioxidant evaluation was performed as in vitro chemical assays (DPPH and ABTS) to confirm the radical scavenging potential of the metabolites. However, the intracellular antioxidant capacity within the macrophages was not assessed in this study. For further study, a cell-based assay using a probe such as DCFH-DA would be a critical next step to confirm if this chemical activity translates to a biologically relevant reduction in reactive oxygen species (ROS) within the target cells.

## 5. Conclusions

This study reveals that LAB-derived metabolites can selectively enhance immune responses in *S. haemolyticus*-infected macrophages, without activating uninfected cells. The metabolites significantly boosted nitric oxide (NO) production and upregulated key inflammatory mediators (iNOS, COX-2, TNF-α, and IL-6) at both gene and protein levels. Crucially, LAB-derived metabolites revealed different profile between different bacterial strain led to different immunomodulatory capacity. These findings demonstrate that LAB metabolites can precisely stimulate immune defenses against bacterial infection. Importantly, this targeted immunomodulation suggests potential for developing LAB-based therapies as alternatives to conventional antibiotics in bovine mastitis treatment, potentially overcoming issues of antibiotic resistance and toxicity. Further research should explore their efficacy in whole-animal models and dairy cattle.

## Figures and Tables

**Table 1:** Primer for qRT-PCR analysis.

Genes	Sense Primer Sequence 5′-3′	Antisense Primer Sequence 5′-3′	References
*iNOS*	TTCCAGAATCCCTGGACAAGC	TGGTCAAACTCTTGGGGTTCG	[20]
*COX-2*	AGAAGGAAATGGCTGCAGAA	GCTCGGCTTCCAGTATTGAG	[21]
*TNF-α*	AGCCCCCAGTCTGTATCCTTC	CATTCGAGGCTCCAGTGAATTCG	[20]
*IL-6*	GCTGGAGTCACAGAAGGAGTG	GCATAACGCACTAGGTTTGCC	[22]
*β-actin*	TGCTGTCCCTGTATGCCTCTG	GCTGTAGCCACGCTCGGTCA	[23]

**Table 2:** Antioxidant activity of LAB-derived metabolites assessed by DPPH and ABTS radical scavenging assays.

Bacterial Strain	Antioxidant Activity	
	DPPH Assay (mg GAE/g Sample)	ABTS Assay (mg TE/g Sample)
*L. casei* TISTR 1340	1.84 ± 0.21 *	12.11 ± 0.99
*L. plantarum* TISTR 2070	1.65 ± 0.15	9.64 ± 0.13
*L. plantarum* AD73	1.59 ± 0.01	14.97 ± 2.52 **
*E. faecalis* TCAN02	1.25 ± 0.36	7.44 ± 0.10

Data presented as mean ± SD of three independent experiments (n = 3). GAE = gallic acid equivalents; TE = Trolox equivalents. * Significantly higher than other strains in each assay (*p* < 0.05) ** Significantly higher than other strains in each assay (*p* < 0.01).

**Table 3:** Key candidate metabolites identified in LAB supernatants and their predominant strain association.

Mode of Detection	Metabolites	Ontology	Predominant Strain(s)
Negative mode	gluconic acid	carbohydrate-derived acids	*L. plantarum* AD73
	glutamic acid	Alpha amino acids and derivatives	*L. plantarum* AD73
	Kaempferol	Flavonoid-O-glycosides	*E. faecalis* TCAN02
	lobaric acid	Depsides and depsidones	*L. casei* TISTR1340
	matairesinol	Phenylpropanoid	*E. faecalis* TCAN02
	Olivetoric acid	Phenolic Acid	*L. plantarum* TISTR2070
	sweroside	secoiridoid glycosides	*L. casei* TISTR1340
Positive mode	Adenosine	Purine nucleosides	*L. plantarum* AD73
	Chrysoeriol	3′-O-methylated flavonoids	*E. faecalis* TCAN02
	Cinnamic acid	Hydroxycinnamic acids	*L. plantarum* AD73
	gastrodin	Phenolic glucoside	*L. plantarum* AD73
	Genistein	Isoflavones	*E. faecalis* TCAN02
	ginsenoside	Triterpenoids	*L. plantarum* TISTR2070
	Isoscopoletin	8-prenylated flavones	*L. plantarum* AD73
	limocitrin	Flavonols	*L. plantarum* TISTR2070
	Luteolin	Flavonoid 8-C-glycosides	*E. faecalis* TCAN02
	phloretin	2′-Hydroxy-dihydrochalcones	*L. casei* TISTR1340
	Rosmarinic acid	Coumaric acids and derivatives	*E. faecalis* TCAN02
	scopoletin	7-hydroxycoumarins	*E. faecalis* TCAN02
	vanillic acid	M-methoxybenzoic acids and derivatives	*L. plantarum* AD73

## Data Availability

The original contributions presented in this study are included in the article/Supplementary Material. Further inquiries can be directed to the corresponding author.

## Supplementary Materials

The following supporting information can be downloaded at: https://www.mdpi.com/article/10.3390/ani15223338/s1, Figure S1: Western blot membrane iNOS protein expression, Figure S2: Western blot membrane COX-2 protein expression, Figure S3: Western blot membrane Beta-actin protein expression.

## Abbreviations

The following abbreviations are used in this manuscript:

ABTS	2, 2-azinobis (3-ethylbenzothiazoline-6 -sulfonic acid)
COX-2	Cyclooxygenase-2
DMEM	Dulbecco’s modified eagle’s medium
DPPH	2, 2-diphenyl-1-picrylhydrazyl
IL-6	Interleukin-6
iNOS	Inducible nitric oxide synthase
MTT	3-(4,5-dimethylthizaol-2-yl)-2,5-diphenyl tetrazolium bromide
RT-qPCR	Quantitative reverse transcription polymerase chain reaction
TNF- α	Tumor necrosis factor-alpha
