# Supplementary material for: Lactic Acid Bacteria Metabolites Modulate Immune Response Against Staphylococcus haemolyticus-Infected RAW264.7 Murine Macrophage: A Novel Approach for Bovine Mastitis
Source: Animals (Basel). 2025 Nov 19;15(22):3338. doi: 10.3390/ani15223338 (PMC12649404; doi:10.3390/ani15223338)
Supplement: Supplementary file 1 [file animals-15-03338-s001.zip › animals-3914723-supplementary.pdf]

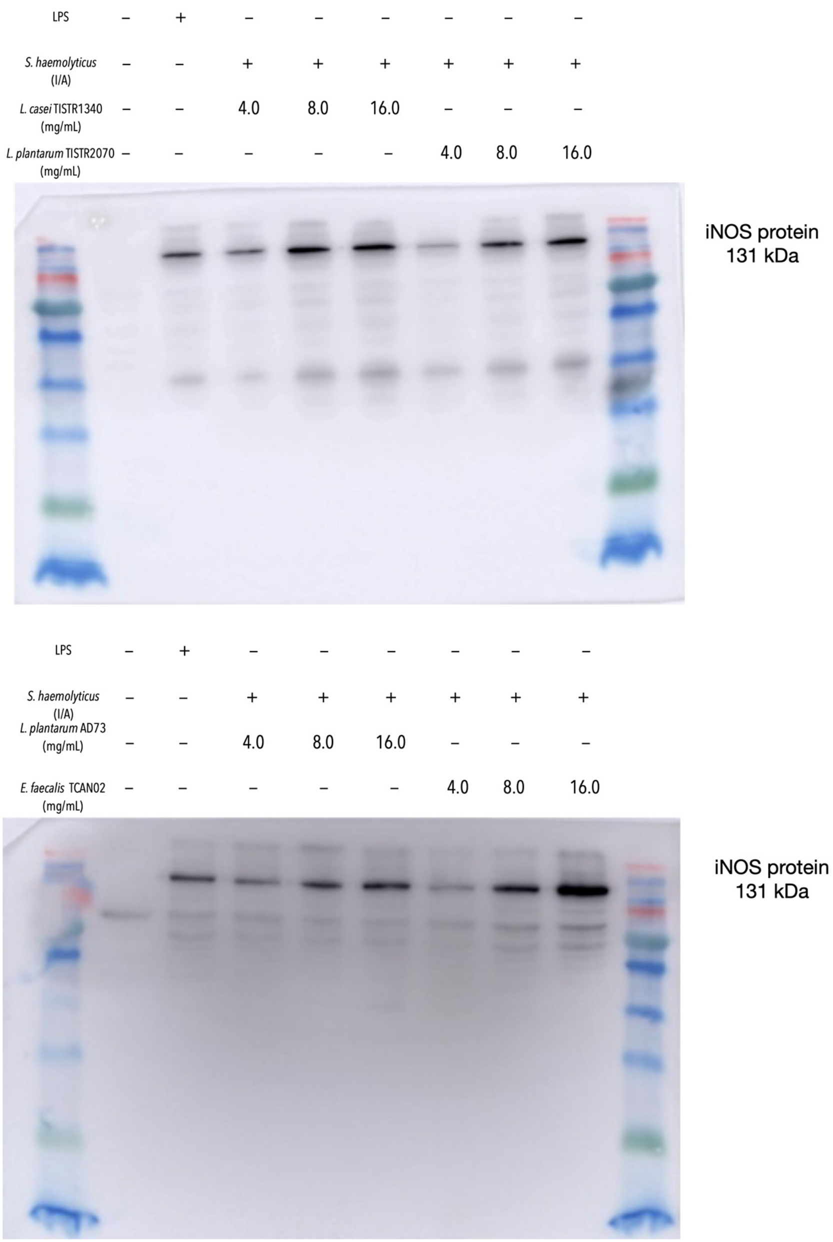

Figure S1. Modulation of iNOS protein expression by LAB-derived metabolites in *S. haemolyticus*-challenged macrophages. RAW264.7 macrophages were co-treated for 24 hours with *Lactobacillus casei* TISTR1340, *L. plantarum* AD73, *L. plantarum* TISTR2070, or *Enterococcus faecalis* TCAN02 metabolites (4.0-16.0 mg/mL) and *S. haemolyticus* (I/A).

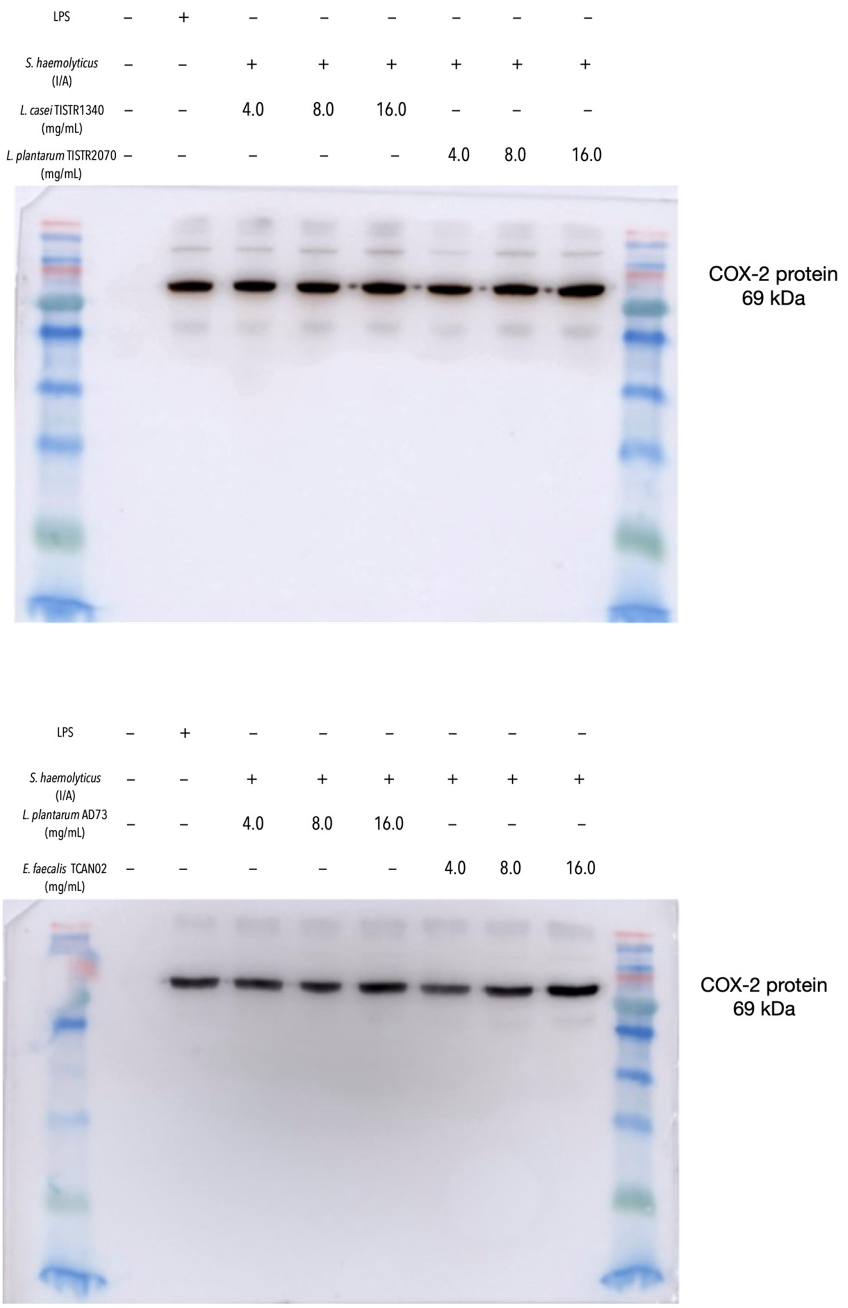

Figure S2. Modulation of COX-2 protein expression by LAB-derived metabolites in *S. haemolyticus*-challenged macrophages. RAW264.7 macrophages were co-treated for 24 hours with *Lactobacillus casei* TISTR1340, *L. plantarum* AD73, *L. plantarum* TISTR2070, or *Enterococcus faecalis* TCAN02 metabolites (4.0-16.0 mg/mL) and *S. haemolyticus* (I/A).

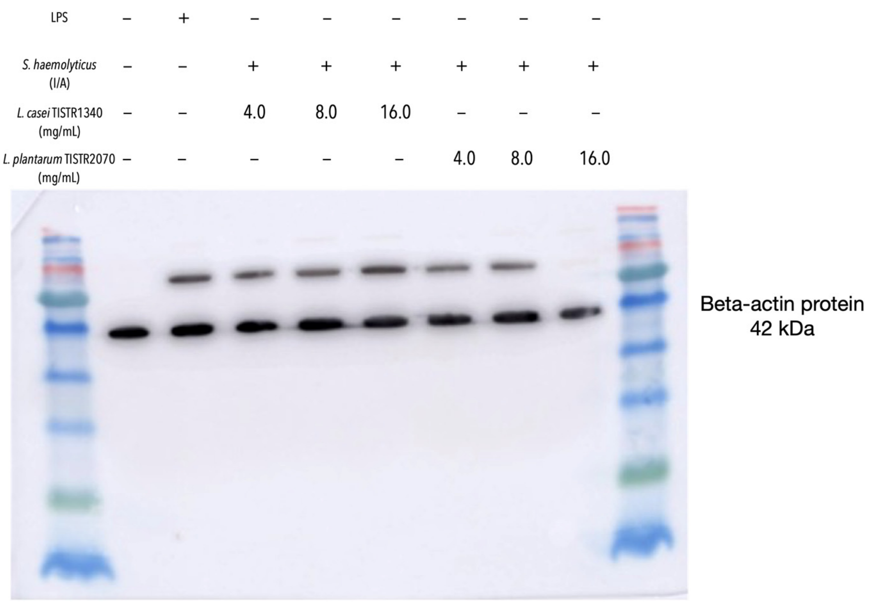

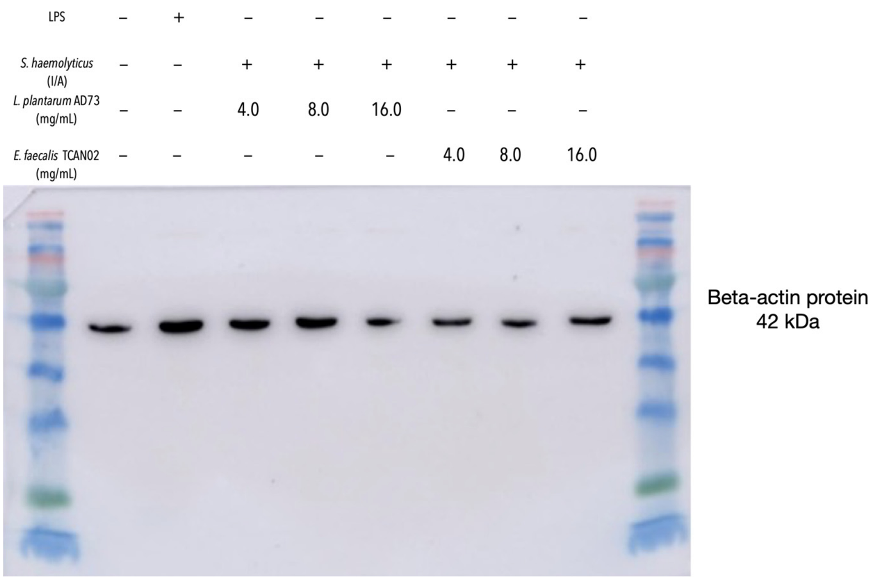

Figure S3. Modulation of Beta-actin protein expression by LAB-derived metabolites in *S. haemolyticus*-challenged macrophages. RAW264.7 macrophages were co-treated for 24 hours with *Lactobacillus casei* TISTR1340, *L. plantarum* AD73, *L. plantarum* TISTR2070, or *Enterococcus faecalis* TCAN02 metabolites (4.0-16.0 mg/mL) and *S. haemolyticus* (I/A).

Migration patterns of TriColor Broad Protein Ladder (3.5–245 kDa) in different electrophoresis conditions.

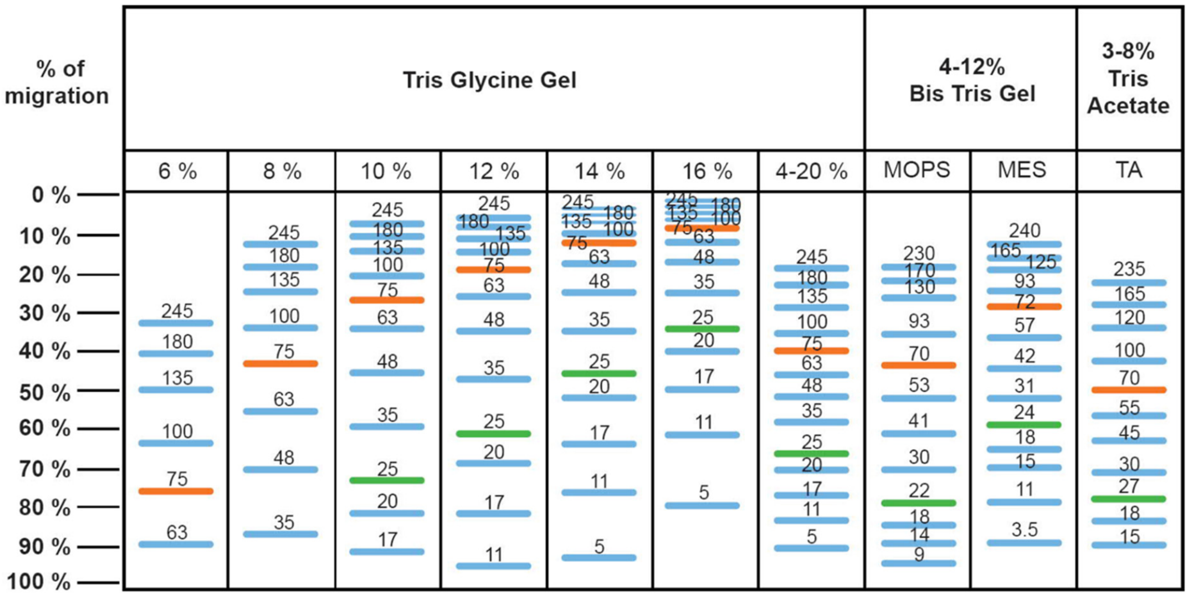

The apparent molecular weight (kDa) of each protein has been determined by calibration against unstained protein standards.
